# Editorial: Role of pigmentation in melanoma

**DOI:** 10.3389/fonc.2022.1084717

**Published:** 2022-11-23

**Authors:** Anna A. Brożyna, Laura Poliseno, Andrzej T. Slominski

**Affiliations:** ^1^ Department of Human Biology, Institute of Biology, Faculty of Biological and Veterinary Sciences, Nicolaus Copernicus University, Toruń, Poland; ^2^ Institute of Clinical Physiology, National Research Council (IFC-CNR), Pisa, Italy; ^3^ Oncogenomics Unit, Core Research Laboratory, Istituto per lo Studio, la Prevenzione e la Rete Oncologica (ISPRO), Pisa, Italy; ^4^ Department of Dermatology, University of Alabama at Birmingham, Birmingham, AL, United States; ^5^ Pathology Laboratory Service, Veteran Administration Medical Center at Birmingham, Birmingham, AL, United States

**Keywords:** melanin, melanogenesis, melanosomes, ROS and drug scavenging, melanosomal pH, melanin unit, melanoma microenvironment, phenotypic shift of melanoma cells

Melanomas are tumors derived from melanocytes. These cells produce melanin pigment, and act as regulators of cutaneous homeostasis, which is disturbed by various stressors (**Figure 1**), a concept formulated almost 30 years ago (1) and further advanced by consideration of melanocyte neuroendocrine properties (3). Melanogenesis is a multistep process that is initiated by tyrosinase-mediated oxidation of tyrosine. Under physiological conditions, melanin synthesized inside melanosomes is transferred from melanocytes to neighboring keratinocytes, creating the functional melanin unit that protects skin cells against ultraviolet-induced damages [(4), Casalou et al.; Slominski et al.]. The protective properties of melanin result from its ability to act as anti-oxidative agent, as well as to bind cations, anions, and other molecules and chemicals. However, such protective properties can be turned to the advantage of melanoma cells, while melanogenesis itself creates toxic intermediates, and pheomelanin is an unstable molecule whose photolysis generates free radicals. These undesirable conditions promote melanomagenesis as well. Pigmentation is in fact associated with worse outcome in metastatic melanoma patients (5) and increased resistance to therapy [(6), Slominski et al.].

**Figure 1:**
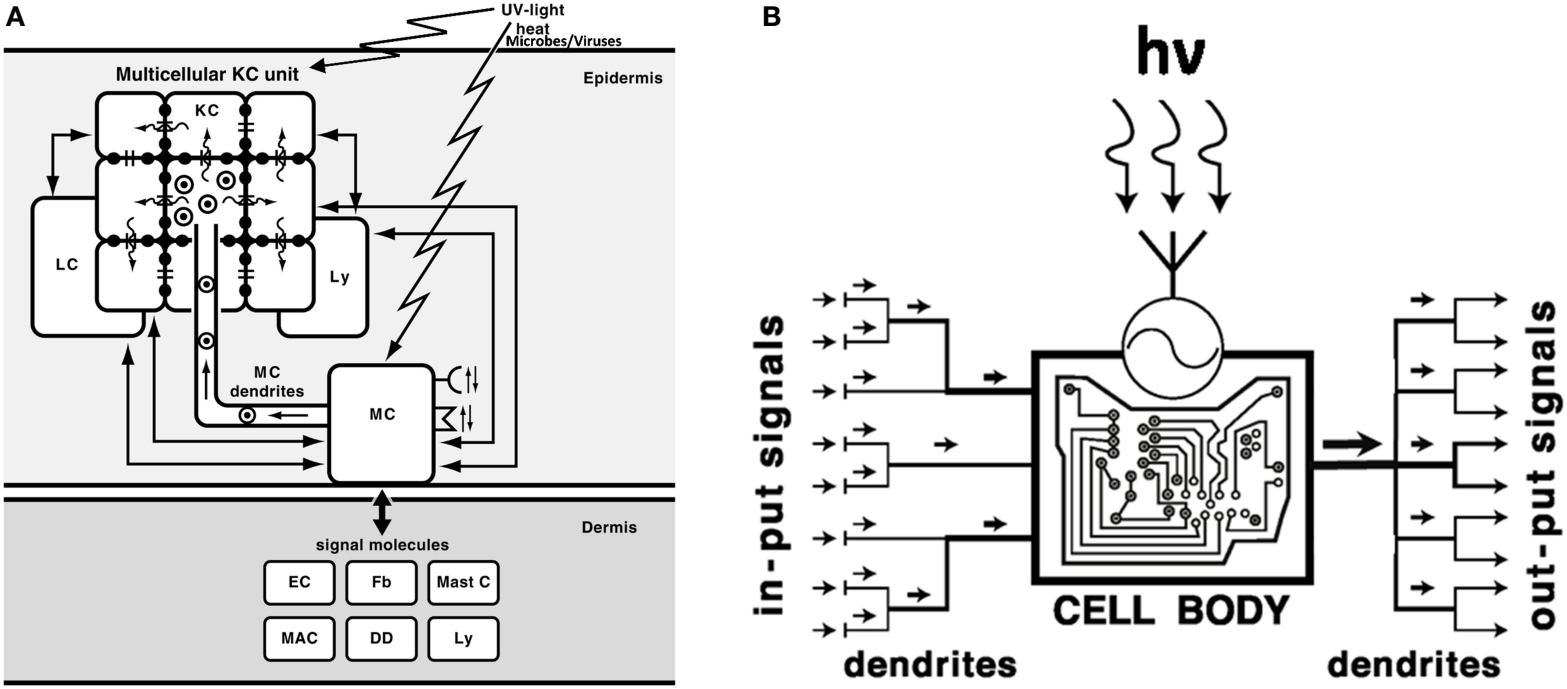
Melanocytes, melanin-producing cells with neuroendocrine capabilities, act as sensory and regulatory cells of the epidermis **(A)** with computing capabilities **(B)**. **(A)**: MC, melanocytes; KC, keratinocytes; LC, Langerhans cells; Ly, lymphocytes; Mast C, mast cells; MAC, macrophages; Fb, fibroblasts; EC, endothelial cells; DD, dermal dendritic cells; 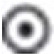, melanosomes; →, direction of melanosomes movement/transfer; =: gap junctions; (
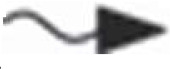
): flow of ions and micromolecules. Reprinted with modifications from (1) with permission from the Elsevier). **(B)**. Melanocytes, after detecting electromagnetic energy of solar radiation or after sensing epidermal disturbances induced by different stress, compute the absorbed energy/information into biologically relevant signals and convey them to multiple effector targets. The UVR induced formation of multiple dendrites not only allows to amplify the biological effect (right output) but also enhances the capability of sensing stress-induced disturbances in the epidermis (left input). hv - a quantum of UV radiation; 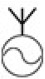
- sensor detecting electromagnetic energy of solar radiation. Reprinted from (2) with permission from the Endocrine Society.

Our Research Topic aims to shed light on the functions of melanosome and melanin under pathological conditions, and on potential new therapeutic strategies with melanin or melanogenesis as molecular targets for melanoma treatment.

Melanogenesis can switch melanocyte and melanoma cell metabolism towards anaerobic, which creates hypoxic conditions and activates hypoxia-inducible factor 1-alpha (HIF-1α) (7). These complex processes are discussed by Slominski et al. The authors also accumulated evidence that melanin can decrease the susceptibility to radio-, chemo-, photodynamic-, and immunotherapies, utilizing its protective properties.

Zippin et al. discuss how the differences in melanosome acidity depend on skin color. Authors point out that melanosome pH determines the ratio of eumelanin vs pheomelanin, with higher eumelanin level in higher pH. It should be noted that this ratio in turn determines the redox state of the cells, since eumelanin and pheomelanin display antioxidative and prooxidative properties, respectively (Slominski et al.). Authors also describe the changes in the expression of genes involved in the regulation of melanosomal pH in melanoma. Finally, they suggest that modulating melanosomal pH can impact on eumelanin, as a reactive oxygen scavenger, hence providing a new therapeutic approach for melanoma patients. These considerations represent a nice extension of the studies performed in the past at Yale University (8).

The close collaboration between melanocytes and keratinocytes as melanin units allows for the proper melanin distribution and maintains skin homeostasis (**Figure 1**) (1). Active melanogenesis affects the functions of keratinocytes, since melanosomes are transferred to surrounding cells (**Figure 1**). And, as Casalou et al. discuss, disturbances in the melanocyte-keratinocytes interaction can be the very early step that triggers the malignant transformation of melanocytes. The changes in the melanin unit can be caused by deregulation of melanocytes to keratinocytes ratio, growth factors expression, and melanosome matrix components involved in melanosome shape and pigmentation of the tumor. The authors also discuss how melanomas can arise either from immature or mature melanocytes, which contributes to their phenotypic heterogeneity.

As discussed by Cabaço et al. melanosomes secreted by melanoma cells affect dermal fibroblasts and stimulate their transformation toward cancer-associated fibroblasts (promoting a tumorigenic and immunosuppressed microenvironment). Moreover, the authors point out that scavenging the reactive oxygen and nitrogen species could initiate the malignant transformation of melanosomes, while the consequent deregulation of melanogenesis can result in the acquisition of invasive properties. Finally, the authors characterize several proteins regulating melanogenesis that are simultaneously involved in melanoma tumorigenesis (e.g. MC1R, TYR, PMEL, RAB27A). The authors conclude that a systematic study of the multifaceted and often non-linear activities of melanin should be undertaken, allowing for the establishment of new therapies based on melanogenesis regulation.

The study of Najem et al. focuses on the effect of melanogenesis on melanoma cells phenotype. The authors show a phenotypic shift under melanogenesis-promoting conditions (high concentration of tyrosine) towards a more aggressive mesenchymal-like, or a senescence-like phenotypes. The former is accompanied by pigmentation fading along with the passages, undifferentiated or mesenchymal-like gene expression profile, loss of E-cadherin expression (a marker of EMT), increased expression of MMPs, and concomitant changes of cell morphology towards undifferentiated cells. The latter is characterized by enlarged morphology, expression and activity of SA-β-Gal, and expression of proinflammatory cytokines. The authors also show that tyrosine-induced mesenchymal-like melanoma cells are not susceptible to MAPK inhibitors, contrary to senescent-like melanoma cells. These studies confirm earlier investigations indicating complex regulatory role of L-tyrosine in melanoma cells (9, 10).

In summary, melanogenesis is an intrinsic feature of melanocytes. This feature can be retained after malignant transformation, but most often melanin synthesis in melanoma cells is deregulated and creates pro-tumorigenic conditions, promoting melanoma growth and invasion. The articles published in this Research Topic provide comprehensive information on the role of melanogenesis and melanin in the initiation and promotion of melanomas, as well as on the complex, diverse functions of pigmentation. This issue also indicates the most crucial research areas in melanoma and melanin biology that should be studied, allowing for future improvements of therapeutic strategies.

## Author contributions

All authors listed have made a substantial, direct, and intellectual contribution to the work and approved it for publication.

## Funding

Writing of the commentary was in part supported by grants from NIH (1R01AR073004-01A1, R01AR071189-01A1), VA merit (1I01BX004293-01A1) and DOD (W81XWH2210689) to ATS. It was also supported by AIRC IG #25694 grant to LP and grant 2014/15/B/NZ4/00751 from National Science Centre, Poland to AAB.

## Acknowledgments

The editors dedicate this Research Topic to Aaron B. Lerner and John M. Pawelek for their contribution to the pigment cell biology.

## Conflict of interest

The authors declare that the research was conducted in the absence of any commercial or financial relationships that could be construed as a potential conflict of interest.

## Publisher’s note

All claims expressed in this article are solely those of the authors and do not necessarily represent those of their affiliated organizations, or those of the publisher, the editors and the reviewers. Any product that may be evaluated in this article, or claim that may be made by its manufacturer, is not guaranteed or endorsed by the publisher.
